# Autoimmune pancreatitis can develop into chronic pancreatitis

**DOI:** 10.1186/1750-1172-9-77

**Published:** 2014-05-21

**Authors:** Masahiro Maruyama, Takayuki Watanabe, Keita Kanai, Takaya Oguchi, Jumpei Asano, Tetsuya Ito, Yayoi Ozaki, Takashi Muraki, Hideaki Hamano, Norikazu Arakura, Shigeyuki Kawa

**Affiliations:** 1 Department of Gastroenterology, Shinshu University School of Medicine, 3-1-1 Asahi, Matsumoto 390-8621, Japan; 2 Endoscopic Examination Center, Shinshu University School of Medicine, 3-1-1 Asahi, Matsumoto 390-8621, Japan; 3 Center for Health, Safety, and Environmental Management, Shinshu University, 3-1-1 Asahi, Matsumoto 390-8621, Japan

**Keywords:** Autoimmune pancreatitis, Chronic pancreatitis, Pancreatic stone, Pancreatic calcification, Relapse, IgG4, IgG4-related disease

## Abstract

Autoimmune pancreatitis (AIP) has been recognized as a distinct type of pancreatitis that is possibly caused by autoimmune mechanisms. AIP is characterized by high serum IgG4 and IgG4-positive plasma cell infiltration in affected pancreatic tissue. Acute phase AIP responds favorably to corticosteroid therapy and results in the amelioration of clinical findings. However, the long-term prognosis and outcome of AIP remain unclear. We have proposed a working hypothesis that AIP can develop into ordinary chronic pancreatitis resembling alcoholic pancreatitis over a long-term course based on several clinical findings, most notably frequent pancreatic stone formation. In this review article, we describe a series of study results to confirm our hypothesis and clarify that: 1) pancreatic calcification in AIP is closely associated with disease recurrence; 2) advanced stage AIP might have earlier been included in ordinary chronic pancreatitis; 3) approximately 40% of AIP patients experience pancreatic stone formation over a long-term course, for which a primary risk factor is narrowing of both Wirsung’s and Santorini’s ducts; and 4) nearly 20% of AIP patients progress to confirmed chronic pancreatitis according to the revised Japanese Clinical Diagnostic Criteria, with independent risk factors being pancreatic head swelling and non-narrowing of the pancreatic body duct.

## Introduction

Autoimmune pancreatitis (AIP) has been recognized as a distinct type of pancreatitis that is possibly caused by autoimmune mechanisms [1-3]. AIP is characterized by pancreatic enlargement and irregular narrowing of the main pancreatic duct (MPD), both of which resemble the imaging features of pancreatic cancer [4-8]. Other prominent features in AIP include high serum IgG4 and IgG4-positive plasma cell infiltration in affected pancreatic tissue, which are used in the serological and pathological diagnosis of AIP, respectively [9-11]. Patients with AIP respond favorably to prednisolone (PSL) therapy from clinical, serological, imaging, and pathological perspectives [12-16]. In 2011, the International Consensus Diagnostic Criteria (ICDC) for AIP enabled diagnosis based on an international standard, and defined type 1 and type 2 AIP according to pathological and clinical findings [17]. Several institutions in Japan and abroad have since confirmed the utility of the ICDC [18-21]. Type 1 AIP corresponds to lymphoplasmacytic sclerosing pancreatitis (LPSP), and type 2 AIP relates to idiopathic duct centric chronic pancreatitis (IDCP) or AIP with granulocytic epithelial lesion (GEL) [11,22-24]. Type 1 AIP is closely associated with increased serum IgG4 concentration and IgG4-bearing plasma cell infiltration in affected tissue [2,9-11]. It is presumed to have specific immunological derangement associated with IgG4, and has now been recognized as a pancreatic manifestation of IgG4-related disease (IgG4-RD). IgG4-RD is a recently established disease concept and novel clinical entity of unknown origin that exhibits multi-organ involvement and abundant infiltration of IgG4-positive cells [2,25-28]. Although the long-term prognosis and outcome of AIP remain unclear, the determination of whether AIP progresses to chronic pancreatitis over time is a significant matter that needs addressing.

We have proposed a working hypothesis that AIP can develop into ordinary chronic pancreatitis resembling alcoholic pancreatitis over a long-term course based on the following published clinical findings: 1) the clinical features of AIP have mainly been reported for an acute state of the disease, in which short-term pancreatic swelling and severe lymphoplasmacytic infiltration were described. These symptoms are unlikely to persist over an extended period of time and are now believed to manifest as different clinical features in a chronic disease state; 2) we have described several patients with AIP who formed pancreatic stones during the course of their disease [29,30], which is a well-known phenomenon confirmed by many other studies [14,29-38]. Since pancreatic stones are a primary characteristic of ordinary chronic pancreatitis, it appears that chronic stage AIP may exhibit symptoms mimicking those of ordinary chronic pancreatitis; 3) approximately 30-40% of ordinary chronic pancreatitis cases are classified as unidentified or idiopathic [39,40], wherein some cases of chronic stage AIP may have been be included; and 4) we measured serum levels of IgG4 in 175 patients with chronic pancreatitis who had been diagnosed before 1995, when the concept of AIP was first proposed, and found high serum IgG4 concentrations in 7.4% of individuals [30]. This indicated that chronic stage AIP may have resulted in the development of ordinary chronic pancreatitis.

In order to investigate and clarify the above points, we evaluated the association between AIP and pancreatic calcification and arrived at the conclusion that AIP could indeed progress to confirmed chronic pancreatitis. In this review article, we describe a series of studies to confirm our working hypothesis.

## Autoimmune pancreatitis and pancreatic calcification

We evaluated the long-term outcomes of patients with AIP and observed that some individuals experienced pancreatic calcification.

### Development of pancreatic calcification

In 1995, Yoshida et al. first proposed a concept of AIP that was considered to be free from calcification [4]. As patients with AIP responded favorably to corticosteroid therapy, the disease was believed to be a non-progressive condition that did not lead to pancreatic stone formation and did not progress to an advanced stage of chronic pancreatitis. Later, it was brought to light that some patients with AIP experienced pancreatic stone formation, pancreatic atrophy, and/or irregular dilatation of the MPD over a long-term course [14,29-38]. Such imaging findings mimicked those of chronic pancreatitis, suggesting that patients with AIP could progress to this state.

We subsequently examined 42 AIP patients who had been followed for at least 12 months (median follow-up period: 54.5 months, range: 13–111 months). Eight patients (19%) developed pancreatic calcification. Because 6 of 11 patients (54.5%) who relapsed had detectable calcification, pancreatic calcification in AIP became significantly associated with relapse patients in comparison with non-relapse patients (p = 0.0019) [29]. Later, we investigated 51 AIP patients who had been followed for a minimum of 2 years (median follow-up period: 72 months, range: 24–178 months), among whom 9 (18%) exhibited pancreatic calcification during the disease course. Pancreatic stone disease was again significantly more frequent in the recurrence group (7/21, 33%) than in the non-recurrence group (2/30, 7%) (p = 0.014) [30]. These studies suggested that pancreatic calcification was closely associated with relapse and that AIP could transform into ordinary chronic pancreatitis after multiple recurrences. The main reason for a presumed association between calcification and relapse may be attributed to recurrent periductal lymphoplasmacytic inflammation, which is a characteristic pathological feature of type 1 AIP [17], that leads to remnant pancreatic duct stricture resulting in pancreatic juice stasis and intraductal calcification. Indeed, our later studies disclosed that pancreatic calcification was significantly associated with pancreatic head swelling and narrowing of both Wirsung’s and Santorini’s ducts in the pancreatic head region [41]. Recurrent parenchymal inflammation with ensuing calcification may also be a contributive factor in AIP relapse.

### Epidemiology of pancreatic calcification

The frequency of pancreatic calcification in AIP ranges from 4% to 40% [14,29,30,32,34-36,38,41,42]. Hart et al. conducted an international investigation on AIP and reported that 46 of 659 patients (7%) demonstrated this condition. Moreover, pancreatic stone formation was significantly more frequent in relapse than in non-relapse subjects (14.4% vs. 4.0%, p < 0.001) [14]. Meanwhile, Nakazawa et al. reported that 3 of 37 patients (8%) experienced pancreatic calcification [32], Suzuki et al. witnessed this in 7 of 50 patients (14%) [34], Takuma et al. observed this in 2 of 50 patients (4%) [36], and Xingang et al. reported stone formation in 7 of 27 patients (26%) [38]. Maire et al. examined 28 patients with type 1 AIP and 16 patients with type 2 AIP, and disclosed that more than one-third of AIP patients developed pancreatic imaging abnormalities of calcification, atrophy, and/or duct irregularities within 3 years of diagnosis [37]. Furthermore, Takada et al. reported that the incidences of osteopontin expression in centroacinar cells as well as chronic pancreatitis with calcification in AIP were significantly greater than those in the normal pancreas (p < 0.05), which implied that AIP had the potential for pancreatic calcification over a long-term clinical course [33]. Although it appears evident that some patients with AIP experience pancreatic stone formation over time, none of the previous studies have addressed the underlying mechanism, risk factors, and effective measures to prevent this complication. Discrepancies in the reported frequencies of pancreatic calcification may be explained by differences in disease activity, observation period, therapeutic method, and especially maintenance therapy.

### Predictors of relapse

Since pancreatic stone formation in AIP was considered to be closely associated with relapse, we turned to means of predicting relapse and treating the disease in its early stage. We investigated the clinical course of a particular female patient with AIP who had experienced several recurrences. Serum levels of IgG4 and immune complexes, as determined by the monoclonal rheumatoid factor method (IC-mRF), tended to rise several months before each recurrence had became clinically apparent [8]. For prediction of further relapses in this patient, it was helpful to periodically check serum values of IgG4 and IC-mRF. IC-mRF performed well in predicting relapse at a cutoff value of 10 μg/dL, with good sensitivity (61.9%), specificity (70.0%), and efficacy (66.7%). The probability of relapse was 60% when IC-mRF was >10 mg/dL and 30% when IC-mRF was <10 mg/dL [30]. Complement factors C3 and C4 have also been reported as useful markers for monitoring disease activity and tissue damage [43].

The relapse rate of AIP has been estimated at 30% to 50% [12,14,29,30,36,44-50]. Relapse patients generally experience 1 or 2 recurrences, although this figure is higher for some. As corticosteroid therapy has been reported to significantly increase the remission rate and decrease the relapse rate of AIP [12,48], it is considered to be the standard treatment for inducing remission [46]. Previous studies have indicated that various factors at diagnosis, including diffuse pancreatic swelling, jaundice, and elevated values for IgG4, soluble IL2 receptor, complement, and immune complexes, are significant predictive factors of relapse [30,36,45,47,48]. Taken together, careful observation of prodromal symptoms, serial measurement of activity markers during the follow-up period, and early intervention and appropriate disease management with corticosteroids are needed to reduce the rate of relapse in AIP.

### Risk factors for pancreatic stone formation

**Figure 1:**
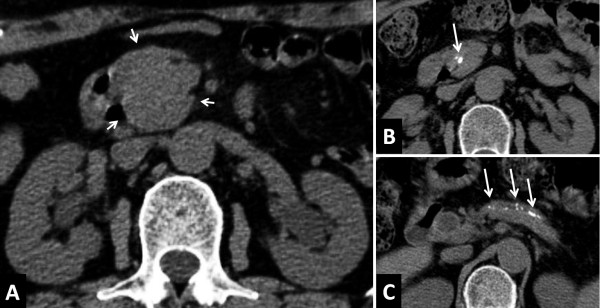
**CT findings in a 67-year-old woman with pancreatic head swelling. (A)** CT at diagnosis in May 2005 showing pancreatic head swelling (arrows). **(B)**, **(C)** CT 27 months later in August 2007 showing pancreatic stone formation and pancreatic atrophy (arrows). Data are reprinted from Ref. [41] with permission from the Journal of Gastroenterology.

**Figure 2:**
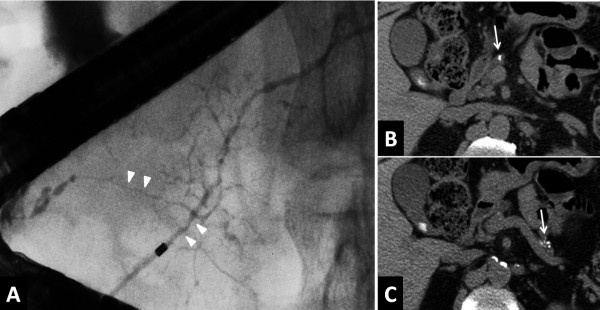
**ERCP and CT findings in a 69-year-old man with narrowing of both Wirsung’s and Santorini’s ducts. (A)** ERCP at diagnosis in April 2001 showing Wirsung and Santorini duct narrowing (arrowheads). **(B)**, **(C)** CT 105 months later in December 2009 showing pancreatic stone formation and pancreatic atrophy (arrow). Data are reprinted from Ref. [41] with permission from the Journal of Gastroenterology.

We have postulated two mechanisms for pancreatic stone formation in AIP: 1) severe tissue injury attributed to the specific inflammatory disease processes; and 2) pancreatic juice stasis due to pancreatic duct narrowing. To examine the risk factors underlying pancreatic stone formation in AIP, we enrolled 69 AIP patients who had been followed for at least 3 years (median follow-up period: 91 months, range: 36–230 months) [41]. During the study period, increased or *de novo* stone formation was seen in 28 patients (28/69, 40.6%), who were defined as the stone-forming group. No stones were observed in 32 patients (32/69, 46.4%), who were considered as the non-stone-forming group. Nine patients who had stones at diagnosis but showed no change during the course of the study were excluded. As a result, approximately 40% of patients with AIP experienced pancreatic stone formation over a long-term course. We compared the two groups with regard to clinical and laboratory findings, as well as to computed tomography (CT) and endoscopic retrograde pancreatography (ERP) results. Univariate analysis revealed no significant differences in clinical or laboratory factors associated with AIP-specific inflammation between the two groups. Relapse was more frequently seen in the stone-forming group, but not significantly. Interestingly, pancreatic head swelling (p = 0.006) (Figure 1A) and narrowing of both Wirsung’s and Santorini’s ducts in the pancreatic head region (p = 0.010) (Figure 2A) at the time of diagnosis were significantly more frequent in the stone-forming group (Table 1). Representative CT findings are shown in Figures 1 and 2 of a 67-year-old woman with pancreatic head swelling due to AIP who experienced pancreatic stone formation and pancreatic atrophy 27 months later (Figure 1B, 1C) and of a 69-year-old male AIP patient with narrowing of both Wirsung’s and Santorini’s ducts who presented 105 months later with pancreatic stones (Figure 2B, 2C). Multivariate analysis identified narrowing of both Wirsung’s and Santorini’s ducts as a significant independent risk factor for pancreatic stone formation (OR 4.4, p = 0.019). Even after corticosteroid therapy, residual pancreatic head swelling and/or narrowing of Wirsung’s and Santorini’s ducts were more frequently found in stone-forming patients in our cohort [41]. These results supported the notion that narrowing of Wirsung’s and Santorini’s ducts in the pancreatic head region led to pancreatic juice stasis and eventual stone formation. On the other hand, there were no significant linkages between stone formation and published well-known activity markers of AIP and no patients developed severe pancreatitis, which suggested that the other hypothesized mechanism, severe tissue injury attributed to the specific inflammatory process of AIP, was poorly associated with pancreatic stone formation. Concerning the distribution of pancreatic stone formation, we noted multiple calcifications in the pancreatic head region in addition to stones in the pancreatic body and tail. Of the 4 patients in the stone-forming group exhibiting duct narrowing in the body and tail of the pancreas, 2 of them had parenchymal pancreatic stones in downstream pancreatic regions [41]. Therefore, some cases of stone formation may be due to factors other than pancreatic juice stasis by stenosis of Wirsung’s and Santorini’s ducts that might be associated with tissue damage attributed to parenchymal inflammation. This study found that moderate alcohol consumption was not a risk factor for pancreatic stone formation, although Hirano et al. reported that high ethanol consumption of >50 g/day increased the risk of stone development and pancreatic atrophy [42]. Further study is needed on the contribution of alcohol consumption to disease pathogenesis.

**Table 1:** **Clinical features, laboratory tests, and pancreatic morphology at diagnosis (From Ref. **[41]**)**

	**Stone-forming**	**Non-stone-forming**	***P *value**
	**group (n = 28)**	**group (n = 32)**	
Clinical features	Median (range)		
Observation period^§^	100 (36–165)	90 (36–230)	0.524
Age	67 (47–84)	64.5 (38–81)	0.543
Sex (M/F)	24/4	22/10	0.140
Alcohol (+/−)	20/8	19/12	0.582
Prednisolone (+/−)	25/3	28/4	1.000
Relapse (+/−)	11/17	6/26	0.093
Laboratory tests			
Amylase	94 (17–431)	86 (22–478)	0.678
IgG	2187 (892–7236)	2183 (1194–5545)	0.686
IgG4	640 (154–2855)	424 (4–2970)	0.916
C3	91 (33–157)	87 (29–199)	0.538
C4	20.1 (7.7–39.7)	21.3 (1.1–38.7)	0.627
sIL2-R	738 (132–2260)	940 (257–4695)	0.130
CIC	5.1 (1.9–40)	5.5 (1.9–27.5)	0.392
Pancreatic morphology at diagnosis			
Pancreatic swelling (by CT)			
Head (+/−)	26/2	20/12	0.006^*^
Body (+/−)	20/8	19/13	0.419
Tail (+/−)	17/11	19/13	1.000
Focal/Segmental-Diffuse	7/21	12/20	0.406
Ductal narrowing in MPD (by ERP)			
Head (+/−)	24/4	22/10	0.140
Wirsung + Santorini (+/−)	21/7	13/19	0.010^*^
Body (+/−)	15/13	19/13	0.795
Tail (+/−)	22/6	24/8	0.770
Focal/Segmental-Diffuse	6/22	11/21	0.390

^**§**^Period from AIP diagnosis to the most recent observation (months).

*P < 0.05.

sIL2-R: soluble interleukin 2 receptor, CIC: circulating immune complex,.

MPD: main pancreatic duct.

Patients with AIP generally respond favorably to PSL therapy [12-16]. However, the efficacy of PSL treatment may vary depending on the stage and activity of AIP [51,52]. We consider that patients with AIP who have residual pancreatic head swelling and/or narrowing of Wirsung’s and Santorini’s ducts after PSL therapy might be at a more advanced stage or active state of AIP. In such cases, treatment with PSL to prevent pancreatic stone formation and progression to chronic pancreatitis may be of limited use, even if the dose is increased. Furthermore, we found no significant linkages between pancreatic stone formation and PSL therapy [41], which suggested that PSL could not halt pancreatic stones from forming. We nonetheless maintain that in early stage AIP, intensive PSL therapy has the potential to prevent pancreatic stone formation as well as the progression to chronic pancreatitis. More detailed analyses of maintenance therapy and follow-up regimens are needed.

### Pancreatic stone formation and pancreatic function: long-term outcomes

With the confirmation of pancreatic stone formation in AIP, it has become essential to investigate whether this complication is associated with eventual pancreatic exocrine or endocrine dysfunction, as seen in ordinary chronic pancreatitis. We compared serum values of amylase and HbA1c at diagnosis of AIP and at 5 and 8 years later in non-stone-forming patients, stone-forming patients, and intraductal stone-forming patients, the latter of which appeared to be at a more advanced stage of stone formation. Although we found no significant differences among the groups, serum amylase and HbA1c levels tended to be abnormally varied in intraductal stone-forming patients as compared with non-stone-forming patients [41]. These results suggest a possible deterioration in pancreatic exocrine or endocrine function in stone-forming patients with AIP over a long-term course, albeit with a different pathophysiology from that of ordinary chronic pancreatitis.

Maire et al. reported that more than one-third of AIP patients experienced pancreatic exocrine or endocrine deficiency in addition to pancreatic calcification, atrophy, and/or duct irregularities, within a 3-year period following diagnosis [37]. Uchida et al. reported in a long-term follow up series (mean follow-up period: 40.8 months, range: 18–130 months) that pancreatic exocrine and endocrine function was not necessarily improved by PSL therapy, which suggested that the efficacy of steroid treatment may be heterogeneous. They speculated that responsiveness to corticosteroids was dependent on the stage and activity of AIP and was probably more effective in the early active phase [51]. Similarly, Hirano et al. uncovered that over a long course of AIP (mean follow-up period: 60 months, range: 12–151 months), glucose tolerance that was too severely compromised did not fully recover, even after PSL therapy. They recommended PSL therapy at an early stage of AIP to preserve insulin secretion [52].

The long-term outcome of pancreatic function in AIP remains unclear, especially with respect to pancreatic stone formation. It is also difficult to assess the natural course of pancreatic exocrine and endocrine function because many patients with AIP are promptly treated with PSL. Further study is needed to address these matters.

## Autoimmune pancreatitis can progress to chronic pancreatitis

Some patients with AIP exhibit severe pancreatic calcification over a long-term course. Pancreatic stones are a primary characteristic of ordinary chronic pancreatitis, which suggests that AIP can progress to a form of chronic pancreatitis that meets the diagnostic criteria for ordinary chronic pancreatitis.

### Serum levels of IgG4 in patients with confirmed chronic pancreatitis

It has been an interesting matter how many patients with advanced stage AIP who demonstrated definite chronic pancreatitis symptoms may have been included with patients having ordinary chronic pancreatitis before the proposal of AIP. We previously reported that more than 60% of AIP patients maintained high serum IgG4 concentrations even after clinical symptoms were resolved [53]. Thus, if advanced stage AIP was previously included in confirmed ordinary chronic pancreatitis, such cases presumably maintained serum IgG4 elevation. We measured the serum values of IgG4 in 175 patients with confirmed chronic pancreatitis who had been diagnosed before 1995, which was the year when the concept of AIP was first proposed, and uncovered raised serum IgG4 in 13 patients (7.4%) (12 men and 1 woman, mean age: 56 years, 9 alcoholic and 4 idiopathic patients). Among them, 3 had initially been diagnosed as having pancreatic cancer, and 1 had just previously been given a diagnosis of AIP after long-term clinical follow-up. The remaining 9 patients showed the typical imaging findings of ordinary chronic pancreatitis, including pancreatic stones and irregular dilation of the MPD [30]. This indicated that advanced stage AIP might have formerly been included in the diagnostic criteria for ordinary chronic pancreatitis, and especially alcoholic pancreatitis.

### Autoimmune pancreatitis can progress to chronic pancreatitis that meets the revised japanese clinical diagnostic criteria for ordinary chronic pancreatitis

**Figure 3:**
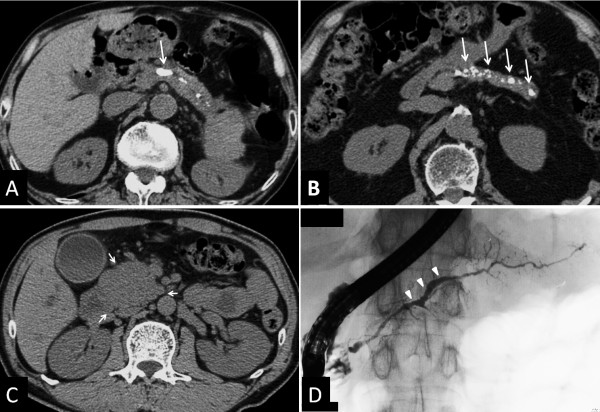
**Images of AIP exhibiting definite chronic pancreatitis, and findings of AIP demonstrating independent risk factors for progression to chronic pancreatitis. (A)** CT image of stones in pancreatic ducts (arrow). **(B)** CT image of multiple or numerous calcifications distributed throughout the entire pancreas (arrows). CT and ERCP findings of AIP demonstrating independent risk factors for progression to confirmed chronic pancreatitis at diagnosis. **(C)** CT finding of pancreatic head swelling at AIP diagnosis (arrows). **(D)** ERP finding of MPD non-narrowing in the pancreatic body at AIP diagnosis (arrowheads). (From Ref. [54]).

After confirming that chronic AIP may have been included in ordinary chronic pancreatitis, we next investigated whether AIP could transform into chronic pancreatitis that specifically met the revised Japanese Clinical Diagnostic Criteria (JCDC) and aimed to clarify the susceptibility factors and underlying mechanisms of AIP progressing to this state [54]. We enrolled 73 patients with AIP who had been followed for at least 3 years (median follow-up period: 88 months, range: 36–230 months). Among them, 16 patients (22%) were confirmed to have chronic pancreatitis according to the revised JCDC for chronic pancreatitis [55], which included 15 patients with definite chronic pancreatitis and 1 patient with probable chronic pancreatitis (Table 2). The major imaging findings in definite cases were stones in pancreatic ducts in 9 patients (Figure 3A) and multiple or numerous calcifications distributed throughout the entire pancreas in 13 patients (Figure 3B). As this study mainly utilized CT and ERP findings, the probable chronic pancreatitis criteria that required MRCP or US (EUS) were excluded [54].

**Table 2:** **Breakdown of diagnostic imaging findings for chronic pancreatitis as determined by the revised Japanese Clinical Diagnostic Criteria for chronic pancreatitis (From Ref. **[54]**)**

**Findings of definite chronic pancreatitis (n = 15)**	**Number**
a. Stones in pancreatic ducts	9
b. Multiple or numerous calcifications distributed in the entire pancreas	13
c. Irregular dilatation of the MPD and irregular dilatation of pancreatic duct branches of variable intensity with scattered distribution throughout the entire pancreas on ERCP	2
d. Irregular dilatation of the MPD and branches proximal to complete or incomplete obstruction of the MPD (with pancreatic stones or protein plugs) on ERCP	2
Findings of probable chronic pancreatitis (n = 1)	
b. Irregular dilatation of pancreatic duct branches of variable intensity with scattered distribution throughout the entire pancreas, irregular dilatation of the MPD alone, or protein plugs on ERCP	1
c. Irregular dilatation of the MPD throughout the entire pancreas plus pancreatic deformity with irregular contour on CT	0

This study did not evaluate MRCP or US (EUS) findings, so the probable chronic pancreatitis findings of (a) and (d), which are judged by these modalities, were excluded.

### Clinical features of autoimmune pancreatitis that meet the revised japanese clinical diagnostic criteria for ordinary chronic pancreatitis: risk factors for progression to chronic pancreatitis

During the course of the above study of 73 patients with AIP, progression to chronic pancreatitis in terms of the revised JCDC was seen in 16 patients (22%), who were defined as the progression group, as compared with the remaining 57 non-progression group patients who did not develop chronic pancreatitis. We compared the two groups with respect to clinical and laboratory factors, as well as to CT and ERP findings. Univariate analysis of clinical features revealed that relapse was significantly more frequent (p = 0.030) in the progression group. We found no significant differences in AIP activity markers between the two groups. In pancreatic imaging findings, pancreatic head swelling was more frequently seen in the progression group, albeit not significantly. Interestingly, MPD narrowing in the pancreatic body was significantly less frequent (p = 0.001) and MPD dilatation at one pancreatic area or more was significantly more frequent (p = 0.001) in the progression group (Table 3). Thirteen patients with AIP having MPD non-narrowing in the pancreatic body contained 8 individuals with dilated duct diameter, all of whom showing simultaneous pancreatic head swelling. Furthermore, multivariate analysis indicated that pancreatic head swelling (OR 12.7, p = 0.023) and MPD non-narrowing in the pancreatic body (OR 12.6, p = 0.001) were significant independent risk factors for the progression to chronic pancreatitis [54]. There were 15 patients with pancreatic head swelling in the progression group, 7 of whom with localized swelling mainly in the pancreatic head (so-called focal type) and 8 of whom with diffuse swelling of the pancreas parenchyma (so-called diffuse type). Similarly, there were 13 patients with MPD non-narrowing in the pancreatic body, 12 of whom with simultaneous pancreatic head swelling. Of these, 4 patients had localized swelling mainly in the pancreatic head (so-called focal type) and 8 patients had diffuse swelling of the pancreas parenchyma (so-called diffuse type). This study uncovered differences between risk factors for pancreatic stone formation and progression to chronic pancreatitis. The reason for this discrepancy might be that the former factors included small pancreatic calculi that did not meet the diagnostic criteria of chronic pancreatitis.

**Table 3:** **Clinical features, laboratory tests, and pancreatic morphology at diagnosis (From Ref. **[54]**)**

	**Progression to CP**	**Non-progression to CP**	***P *value**
	**(n = 16)**	**(n = 57)**	
Clinical features	Median (range)		
Observation period^§^	102 (37–165)	87 (36–230)	0.522
Age	66.5 (48–75)	65 (38–84)	0.989
Gender (M/F)	13/3	43/14	0.748
Alcohol (+/−)	6/10	29/28	0.405
PSL (+/−)	13/3	50/7	0.681
PSL maintenance therapy (+/−)	10/6	41/16	0.542
Relapse (+/−)	8/8	12/45	0.030^*^
Laboratory tests			
IgG	2140 (1166–3861)	2227 (892–7236)	0.509
IgG4	421 (146–1845)	663 (4–2970)	0.267
C3	100 (52–122)	98 (29–218)	0.551
C4	21.8 (12.4–37.7)	21.1 (1.1–47.3)	0.495
sIL2-R	726 (132–1845)	892 (257–4695)	0.053
CIC	5 (1.9–13.9)	5.7 (1.4–40)	0.219
Pancreatic morphology at diagnosis			
Pancreatic swelling (by CT)			
Head (+/−)	15/1	41/16	0.096
Body (+/−)	12/4	36/21	0.553
Tail (+/−)	10/6	37/20	1.000
Level 1/Level 2^Φ^	8/8	30/27	1.000
Ductal narrowing in MPD (by ERP)			
Head (+/−)	13/3	44/13	1.000
Wirsung & Santorini (+/−)	11/5	34/23	0.573
Body (+/−)	3/13	37/20	0.001^*^
Tail (+/−)	12/4	42/15	1.000
Level 1/Level 2^Ψ^	6/10	17/40	0.558
Ductal dilatation in MPD (+/−)	9/7	7/50	0.001^*^

^**§**^Period from AIP diagnosis to the most recent observation (months).

^**Φ**^Swelling was classified as level 1 (diffuse swelling) or level 2 (focal/segmental swelling) as defined by the International Consensus Diagnostic Criteria for Autoimmune Pancreatitis.

^**Ψ**^Pancreatic duct narrowing was classified as level 1 (long [segmental/diffuse] or multiple strictures) or level 2 (focal narrowing) as defined by the International Consensus Diagnostic Criteria for Autoimmune Pancreatitis.

*P < 0.05.

CP: chronic pancreatitis, PSL: prednisolone, sIL2-R: soluble interleukin 2 receptor,

CIC: circulating immune complex, MPD: main pancreatic duct.

### Period and ratio of progression to chronic pancreatitis

**Figure 4:**
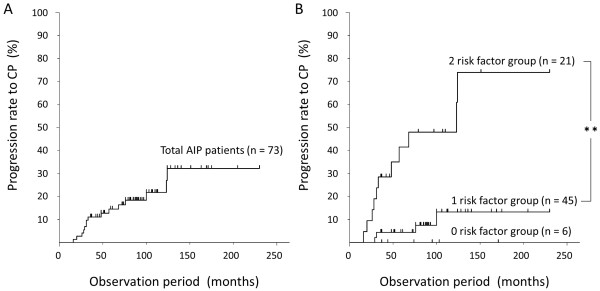
**Progression rate for AIP to chronic pancreatitis. (A)** Kaplan-Meier analysis of the progression rate to confirmed chronic pancreatitis in 73 patients with AIP. **(B)** Kaplan-Meier analysis of the progression rate to confirmed chronic pancreatitis in AIP based on the risk factors of pancreatic head swelling and MPD non-narrowing in the pancreatic body. Comparison of zero risk factor (n = 6), 1 risk factor (n = 45), and 2 risk factor (n = 21) groups. (From Ref. [54]). ^**^P < 0.001 (log-rank test). *CP: chronic pancreatitis.*

We witnessed the median time from AIP diagnosis to chronic pancreatitis to be 33 months (range: 16–124 months) in the above study. Kaplan-Meier testing revealed that the progression rate to chronic pancreatitis was 10% at 36 months, 20% at 100 months, and 30% at 124 months (Figure 4A). Advancement to chronic pancreatitis was not observed after 124 months, suggesting that the window for disease development was within 10 years of follow-up. Next, we performed stratification analysis for AIP progression to chronic pancreatitis using the two risk factors identified by multivariate analysis of pancreatic head swelling and MPD non-narrowing in the pancreatic body. Kaplan-Meier testing was performed on 3 groups: the zero risk factor group (6 patients), the 1 risk factor group (45 patients), and the two risk factor group (21 patients). No AIP patients progressed to chronic pancreatitis in the zero risk factor group. The 2 risk factor group showed a significantly higher progression rate (30% at 3 years and 60% at 10 years) than did the 1 risk factor group (p < 0.001, log-rank test) (Figure 4B) [54]. We could therefore recommend careful follow-up of patients with AIP having these 2 risk factors to prevent progression to chronic pancreatitis.

## Mechanism of autoimmune pancreatitis progressing to chronic pancreatitis

**Figure 5:**
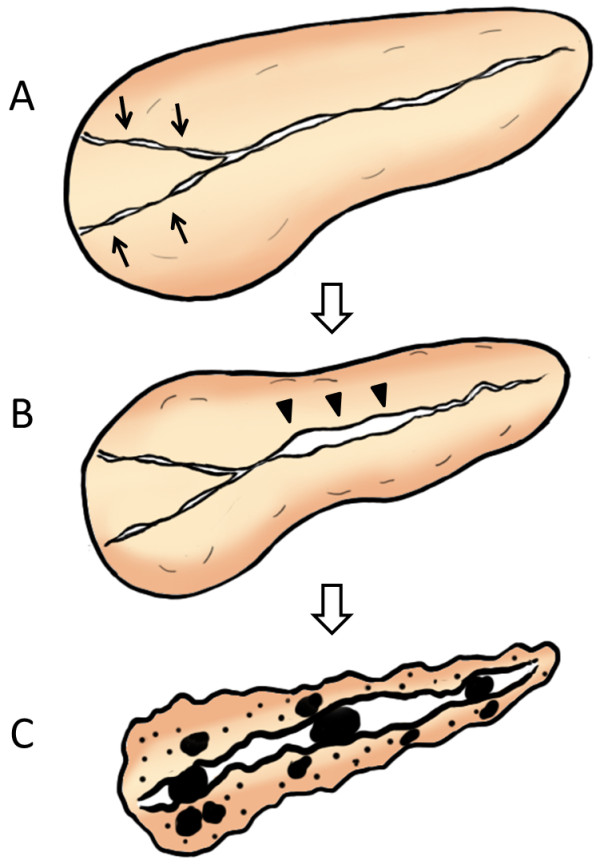
**Sequential progression mechanism of AIP to confirmed chronic pancreatitis. (A)** Narrowing of both Wirsung’s and Santorini’s ducts (arrows) by pancreatic head swelling causes pancreatic juice stasis in the upstream pancreatic duct. **(B)** Pancreatic juice stasis results in increased intra-pancreatic duct pressure that is resistant to typical AIP-specific MPD narrowing in the pancreatic body region, leading to MPD non-narrowing in this region (arrowheads). **(C)** In concert with relapse, these events finally result in severe calcification. (From Ref. [54]).

Based on our cumulative study outcomes, we have proposed the hypothesis of a sequential progression mechanism of AIP to chronic pancreatitis: many cases of AIP include pancreatic head swelling during the acute stage, some of which lead to long-standing narrowing of both Wirsung’s and Santorini’s ducts in this region, which may then cause pancreatic juice stasis in the upstream pancreatic duct (Figure 5A). This might later induce increased intra-pancreatic duct pressure that is resistant to typical AIP-specific MPD narrowing in the pancreatic body, causing MPD non-narrowing in this region (Figure 5B). In concert with relapse, these events may ultimately result in severe calcification of the entire pancreas and pancreatic atrophy (Figure 5C) [54].

## Limitations and future perspectives

There are several limitations to this review. Specifically, our earlier study designs included a limited number of patients and were mainly retrospective in nature. They also applied the revised JCDC for chronic pancreatitis with a strong emphasis on imaging findings, and the subjects enrolled were primarily patients with type 1 AIP. Due to our reliance on imaging findings, more detailed analysis of physical findings, exocrine and endocrine dysfunction, and pathological findings in advanced stage AIP showing severe pancreatic calcification is needed.

## Conclusions

Over the last decade, it has been become apparent that some patients with AIP can experience pancreatic stone formation, pancreatic atrophy, and/or irregular dilatation of the MPD over a long-term course, which may shed light on the matter of whether or not AIP can progress to confirmed chronic pancreatitis. This cumulative study has clarified the following points: 1) pancreatic calcification in AIP is closely associated with disease recurrence; 2) advanced stage AIP might have earlier been included in ordinary chronic pancreatitis; 3) approximately 40% of patients with AIP experience pancreatic stone formation over a long-term course, for which a primary risk factor is narrowing of both Wirsung’s and Santorini’s ducts; and 4) nearly 20% of AIP patients progress to confirmed chronic pancreatitis according to the revised JCDC, with independent risk factors of pancreatic head swelling and MPD non-narrowing in the pancreatic body. Finally, AIP can lead to severe pancreatic stone formation and progress to confirmed chronic pancreatitis over a long-term period, which may be most presumably caused by disease recurrence and pancreatic juice stasis. From these observations, we believe that it is necessary for patients with AIP to maintain a carefully devised follow-up regimen to prevent possible progression to chronic pancreatitis.

## Abbreviations

AIP: Autoimmune pancreatitis; MPD: Main pancreatic duct; PSL: Prednisolone; ICDC: International Consensus Diagnostic Criteria; LPSP: Lymphoplasmacytic sclerosing pancreatitis; IDCP: Idiopathic duct centric chronic pancreatitis; GEL: Granulocytic epithelial lesion; IgG4-RD: IgG4-related disease; IC-mRF: Immune complexes, as determined by the monoclonal rheumatoid factor method; CT: Computed tomography; ERP: Endoscopic retrograde pancreatography; OR: Odds ratio; JCDC: Japanese Clinical Diagnostic Criteria; MRCP: Magnetic resonance cholangiopancreatography; US: Ultrasonography; EUS: Endoscopic ultrasonography; sIL2-R: Soluble interleukin 2 receptor; CIC: Circulating immune complex; CP: Chronic pancreatitis.

## Competing interests

The authors declare that they have no competing interests.

## Authors’ contributions

MM and SK conceived the project and wrote the manuscript. All authors were involved in recruitment of patients and diagnosis of AIP, actively exchanged ideas, and approved the final version.
